# Relevance of Toxicity Assessment in Wastewater Treatments: Case Study—Four Fenton Processes Applied to the Mineralization of C.I. Acid Red 14

**DOI:** 10.1155/2015/945489

**Published:** 2015-06-08

**Authors:** Rajaa Idel-aouad, Manuel Valiente, Carmen Gutiérrez-Bouzán, Mercè Vilaseca, Abdlrani Yaacoubi, Boumediene Tanouti, Montserrat López-Mesas

**Affiliations:** ^1^Centre Grup de Tècniques de Separació en Química (GTS), Química Analítica, Departament de Química, Universitat Autònoma de Barcelona, 08193 Bellaterra, Spain; ^2^Institut d'Investigació Tèxtil i Cooperació Industrial de Terrassa (INTEXTER), Universitat Politècnica de Catalunya BarcelonaTech (UPC), Colom 15, 08222 Terrassa, Spain; ^3^Laboratory of Organic Applied Chemistry, Unit of Environmental and Experimental Methodology, Faculty of Sciences Semlalia, University of Cadi Ayyad, 2390 Marrakech, Morocco; ^4^Laboratory of Condensed Matter and Environment, Faculty of Sciences Semlalia, University of Cadi Ayyad, 2390 Marrakech, Morocco

## Abstract

Fenton and Fenton-like processes, both in homogeneous and heterogeneous phases, have been applied to an aqueous solution containing the dye AR 14 in order to study the mineralization and toxicity of the solutions generated after color elimination. The mineralization of AR 14 occurred slower than the decolorization. The Microtox analysis of the treated solutions showed low toxicity intrinsic to the chemicals used in the process rather than the degradation products obtained after the treatment of the dye solution. The dye degradation for the Fenton oxidation process was initially faster than for the Fenton-like process but after a short time, the four processes showed similar degradation yields. All processes have shown good results being the heterogeneous process the most convenient since the pH adjustment is not necessary, the catalyst is recovered and reused and the generation of contaminated sludge is avoided.

## 1. Introduction

The growing industrialization has resulted in a dramatic increase of water pollution which has led to a greater environmental concern; that is, the textile industry can release about 15% of the dye into the effluents during the textile dyeing. The discharge of these effluents into natural stream and rivers poses severe problems, because the presence of small amounts of dyes (below 1 ppm) is clearly visible and influences the water environment considerably [1]. Thousands of scientific studies have been focused on wastewater remediation and a wide variety of treatments have been proposed such as biological and traditional physical techniques [2], advanced oxidation processes (AOPs), and electrochemical methods [3–5]. One of the most important AOPs is the oxidizing process based on the homogeneous Fenton reagent (Fe^2+^/H_2_O_2_) [6, 7]. The mixture of ferrous ion and hydrogen peroxide in the Fenton's reagent generates hydroxyl radical (OH^•^) in situ [8], according to

(1) $$\begin{array}{c} Fe^{2+}+H_{2}O_{2}⟶Fe^{3+}+OH^{-}+OH^{•} \end{array}$$

The Fe^3+^ produced in this reaction reacts with H_2_O_2_ to regenerate Fe^2+^ as shown in (2) and (3). On the other hand, instead of generating Fe^3+^, the ion can be directly added to the solution, replacing the Fe^2+^, in a process called Fenton-like (Fe^3+^/H_2_O_2_):

(2) Fe3++H2O2⟶FeOOH2++H+

(3) FeOOH2+⟶Fe2++HO2•

The impossible recuperation of the catalyst, a narrow active range of pH (3–5), and the need of removing the iron compounds from the purified effluent are disadvantages that strongly restrict the possible application of this process [9]. A solution to the problem could be the use of heterogeneous solid Fenton catalysts, such as clays, silicas, and zeolites whose recovery from water and regeneration are easy to perform [10–12]. These materials have demonstrated being effective catalysts for the oxidative breakdown of phenol [13] and some textile dyes in water [14] among others.

Usually these studies include a conscientious analytical study on pollutants determination or even on degradation mechanisms, but the evaluation of effluent toxicity is generally not carried out. However, the evaluation of wastewaters should include toxicity tests to complement the chemical characterization. Toxicity tests have gained importance since many substances are toxic to living beings at levels below chemical detection limits. Then, a combined approach using instrumental methods for chemical analysis and bioassays for ecotoxicological testing would be extremely important to hazard/risk assessment of wastewater treatment plant discharges [15]. These tests can be performed with simple equipment based on the use of a bacterium, alga, or crustacean. Despite their simplicity, speed, and low cost, they provide very useful information [16]. Several toxicity methods can be used, with Microtox being one of the most popular ones.

Microtox assay is a biosensor-based measurement system for toxicity evaluation, mainly used for water and wastewater assessment [17, 18]. It is widely accepted as a standard bioassay for rapid and accurate toxicity monitoring [19, 20] because it provides a rapid and effective way to detect toxicity caused by a wide range of organic and inorganic contaminants.

In this work, the Microtox toxicity test is applied to evaluate the toxic effect of different treatments on the mineralization of the acid dye C.I. Acid Red 14 (AR 14) in aqueous medium (Fenton and Fenton-like processes in homogeneous and heterogeneous phases). This dye has been the one chosen as a model pollutant because acid dyes are the most widely used in many industrial sectors, such as alimentary, pharmaceutical, chemical, and textile ones.

AR 14 is classified as nontoxic, even food grade, but at the concentrations required to give intense color, it can cause some unpleasant reactions, such as lung, skin, and eye irritation and brain fog. In fact, safety sheets provide a long list of safety precautions [21]. Also, a possible link between the consumption of this artificial dye and an increased hyperactivity in children has been stablished [22]. In this concern as for precautionary principle, the European regulatory community reduced the acceptable daily intake for food colorings.

## 2. Experimental

### 2.1. Dye and Chemicals

**Figure 1:**
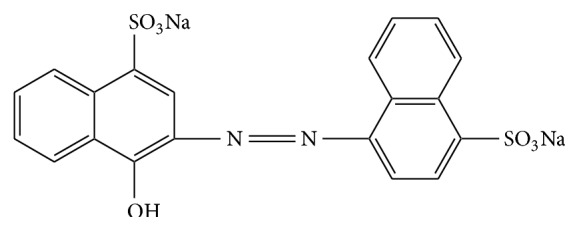
Chemical structure of AR 14.

Basovit Red 440 E (C_20_H_14_O_7_N_2_S_2_Na_2_, Figure 1), which is the commercial name of C.I. Acid Red 14 [20], was purchased from Sigma Aldrich (Spain) and used without further purification. It is chemically classified as monoazo, with bluish red color, molecular weight of 502.44 g/mol, and *λ*_max_ of 516 nm. The salts (NH_4_)_2_Fe(SO_4_)_2_·6H_2_O and Fe(NO_3_)_3_·9H_2_O were purchased from Panreac (Spain) and used for the Fenton and Fenton-like processes, respectively, as well as for the catalysts preparation. H_2_O_2_ (35% w/w) was from FLUKA (Spain). H_2_SO_4_ and NaOH (0.1 M) were from Scharlau (Spain) and used to adjust the pH. All the solutions were prepared with MilliQ or double deionized water. The catalysts were prepared from commercially available ZeolystTM (zeolite Y), produced by Zeolyst International, USA. The zeolite Y-type CBV 712, in its ammonium cation form, has a surface area of 730 m^2^/g, a SiO_2_/Al_2_O_3_ molar ratio of 12, and a Na_2_O weight % of 0.05. It is well known that the unit cell size of Y zeolite is about 24.33–24.35 Å (not provided by the manufacturer) [23, 24].

### 2.2. Catalysts Preparation and Characterization

Heterogeneous Fenton catalyst Fe(III)-zeolite Y and Fe(II)-zeolite Y were prepared from zeolite Y through a cation exchange process [6]. For preparing the Fe(III)-zeolite Y, the zeolite was ion exchanged with an excess of 0.05 M Fe(NO_3_)_3_·9H_2_O for 1 h at room temperature. For the preparation of Fe(II)-zeolite Y, zeolite Y was ion exchanged with an excess of 0.05 M of (NH_4_)_2_Fe(SO_4_)_2_·6H_2_O for 1 h under N_2_ atmosphere at ambient temperature to avoid the oxidation of the iron. The catalyst already prepared was then filtered, thoroughly washed three times with distilled water, and dried in an oven under air at 60°C overnight. Characterization of the heterogeneous catalysts was carried out by X-ray diffraction (XRD) measured with a Philips XPert MPD using Cu K*α* radiation at 40 kV and 30 mA with a scanning speed of 2*θ* = 2 S^−1^. The total amount of iron loaded onto the Fe(III)-zeolite Y and Fe(II)-zeolite Y was determined by using Portable X-Ray Fluorescence equipment (FP-XRF, Alpha-6500R, Innov-X Systems, Inc., Woburn, MA, USA), which consists in a tube-type energy dispersive instrument with a tungsten cathode and a silver anode that can generate X-rays in the energy range 10 to 40 keV and 10–50 *μ*A.

### 2.3. Fenton-Like and Fenton Experiments

The homogeneous Fenton-like and Fenton processes were carried out by adding, respectively, the needed amount of Fe(NO_3_)_3_·9H_2_O or (NH_4_)_2_Fe(SO_4_)_2_·6H_2_O to 100 mL of solution of the dye to obtain 2.17 mM of iron ions. Five minutes later, 750 *μ*L of H_2_O_2_ (35%) was added and the time for the kinetic studies started to count.

For the heterogeneous process, Fe-zeolite Y prepared as previously described was used at the optimized conditions set in our previous study [25] and as follows: initial dye concentration of 50 ppm, concentration of H_2_O_2_ 8.7 mM, temperature 80°C, and initial pH value close to 5.96 (natural pH of the solution). These values were adopted for comparing the different homogeneous and heterogeneous Fenton and Fenton-like processes. Because the amount of iron loaded to each of the different catalysts prepared was different, the amount of the catalysts added to the dye solution was always calculated in order to ensure the same amount of iron ions added to the solution (approximately 2.5 and 15 g/L for Fe(III)-zeolite Y and Fe(II)-zeolite Y, resp.).

Dye oxidation experiments were carried out in 250 mL thermostatic glass reactor equipped with a magnetic stirrer, thermometer, and pH electrode.

### 2.4. Kinetics of Decolorization

At preselected time intervals, 1 mL of sample was taken from the glass reactor, filtrated through a 0.45 *μ*m Millipore filters, and analyzed or frozen until being analyzed (procedure that was corroborated to stop the reaction). Filters were tested in order to check if the dye was absorbed on them and no concentration change was observed. The degree of decolorization of the dye was spectrophotometrically followed measuring the absorbance using a UNICAM UV-Visible spectrophotometer selected at the wavelength with highest absorbance, *λ*_max_ = 516 nm. The dye concentration was quantified through a calibration curve according to the Lambert-Beer law. The degree of decolorization was calculated using

(4) $$\begin{array}{c} \text{Decolourization efficiency}\left(\;\%\;\right)=\frac{C_{0}-C_{t}}{C_{0}}\times 100, \end{array}$$

where *C*_0_ and *C*_*t*_ are the concentrations (mg/L) of the dye at time 0 and *t*, respectively.

In order to model the kinetics of the decolorization, the pseudo-first-order rate equation was used:

(5) $$\begin{array}{c} -Ln\frac{C_{t}}{C_{o}}=Kt, \end{array}$$

where *C*_*t*_ is the dye concentration at time *t* (mol/L), *C*_*o*_ the dye concentration at initial time (*t* = 0), *k* the pseudo-first-order rate constant of consumption (min^−1^), and *t* the time of reaction in minutes.

An ICP-OES, Iris Intrepid II (Thermo Electron, USA), was used for the quantitative determination of iron ions in solution. Sample introduction was performed by a peristaltic pump (1.5 mL/min) connected to a Meinhard nebulizer and then to a cyclone spray chamber. The nebulizer gas was 21 mL Ar/min. These parameters were optimal conditions for this instrument. For every ten samples measured, a QC standard of iron was performed.

### 2.5. Mineralization and Toxicity Tests

Total organic carbon (TOC) concentration was carried out in a TOC analyzer (Shimadzu TOC-5050A) to evaluate the mineralization of the dye after the four processes. The mineralization efficiency of the dye was calculated using

(6) Mineralization  efficiency%=TOC0−TOCtTOC0×100,

where TOC_*t*_ is the value of TOC obtained at time *t* and TOC_0_ corresponds to the initial value of TOC.

The aqueous dye solutions of Acid Red 14 after the four oxidation processes were analyzed by bioluminescence assay in a Microtox 500 apparatus from MICROBICS. Tests were performed using the luminescence bacterium* Vibrio fischeri*, method of lyophilized bacteria [26], according to the international standard ISO 11348-3:2007. The Microtox organism* Vibrio fischeri* (*Photobacterium phosphoreum*) is a bioluminescent organism that produces light as a by-product of its normal metabolism and has demonstrated the highest sensitivity across a broad range of toxicants. The Microtox Acute Toxicity Test measures the relative toxicity of the water sample by recording the light output of the luminescent bacteria before and after exposure to the sample and statistically processes raw data to produce reports on the toxicity of the sample. The level of toxicity is proportional to the inhibition of light production [27]. Luminescent bacteria and all reagents required for the assay were obtained from MICROBICS. The commercially lyophilized bacteria were reconstituted just prior to analysis and incubated with the corresponding sample at 15°C following the mentioned protocol. Effects were calculated as percent of inhibition and based on the decrease of bioluminescence in the samples related to the control solution. Toxicity is expressed as the concentration of compound that produces 50% of bioluminescence inhibition (EC_50_). When the sample is constituted by a mixture of compounds or contains unknown compounds the result is expressed as percentage of sample that produces EC_50_. In order to make a better interpretation of the result, the toxicity impact index (TII_50_), also called Equitox/m^3^, is used and defined as in (7) where TII_50_ is directly proportional to the toxicity of the sample:

(7) $$\begin{array}{c} TII_{50}=\frac{100}{EC_{50}}. \end{array}$$

All the analyses were carried out in duplicate and results are expressed as the average ± RSD (95% of confidence level).

## 3. Results and Discussion

### 3.1. Catalysts Characterization

**Figure 2:**
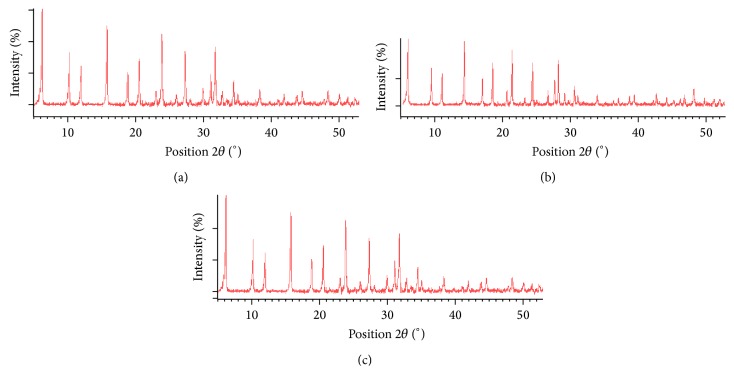
XRD patterns of (a) Fe(II)-Y zeolite, (b) Fe(III)-Y zeolite, and (c) zeolite.

Fe(III)-zeolite Y and Fe(II)-zeolite Y were checked by powder X-ray diffraction. This analysis showed that the catalyst had a typical USY zeolite structure and no amorphous material was present (Figure 2). Introduction of iron cations into USY zeolite via ion exchange had virtually no effect on its crystalline structure. The total amount of iron loaded onto Fe(III)-zeolite Y and Fe(II)-zeolite Y was determined by X-ray fluorescence, found to be, respectively, 12.12 × 10^−2^ and 2.02 × 10^−2^ g of Fe/g zeolite Y.

### 3.2. Acid Red 14 Decolorization by Heterogeneous and Homogeneous Fenton Processes

**Figure 3:**
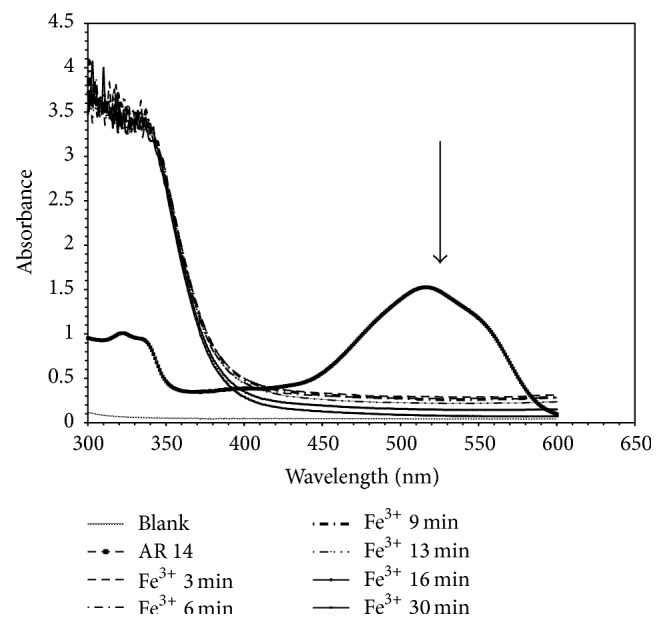
UV-Visible spectra changes of Acid Red 14 during Fenton-like process (Fe^3+^/H_2_O_2_).

**Figure 4:**
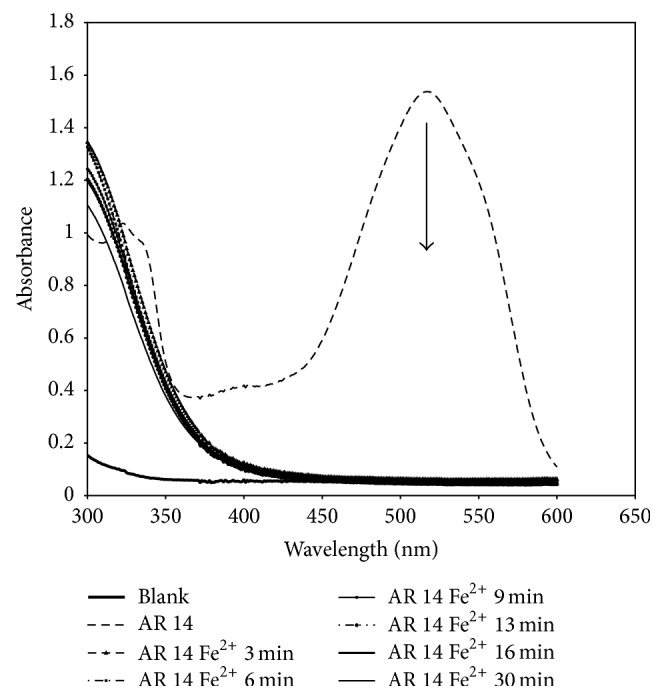
UV-Visible spectra changes of Acid Red 14 during Fenton process (Fe^2+^/H_2_O_2_).

It was verified that for both homogeneous processes, Fenton and Fenton-like, the simple addition of the salts used for the solution of the dye in the absence of H_2_O_2_ had no effect on the AR 14 spectra. Then, when the hydrogen peroxide was added, the color of the solution changed from clear red to turbid brown within 30 s. As the reaction progressed, the dark color of the solution was changed to light yellow and after 30 min was clear and almost colorless indicating that the reaction was complete. Figures 3 and 4 show, respectively, a typical time-dependent UV-Vis spectrum of AR 14 under Fenton and Fenton-like processes. As it is seen, the absorption of the main band with a maximum at 516 nm decreased to finally disappear indicating that the AR 14 had been degraded. No new absorption bands appear in either the visible or ultraviolet regions.

**Figure 5:**
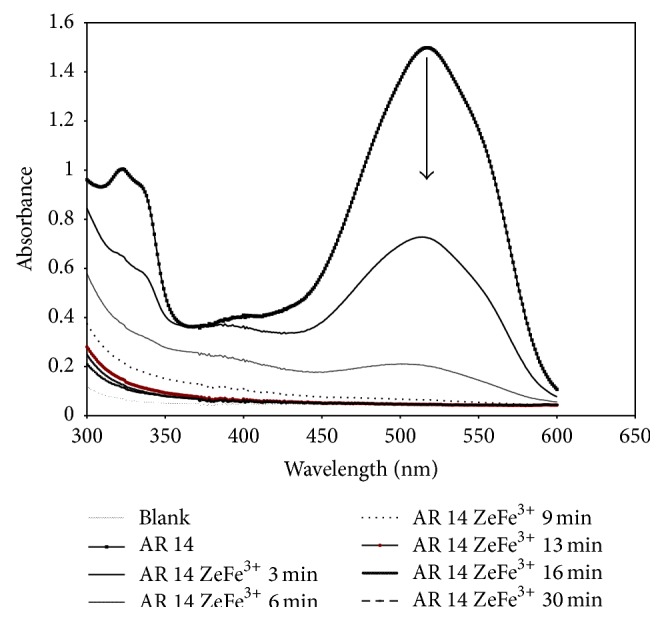
UV-Visible spectra changes of Acid Red 14 during Fe(III)-Y zeolite/H_2_O_2_ process.

AR 14 has negative sulfonate groups, which are repelled by the negatively charged zeolite surface which induces a relatively low adsorption capacity for natural zeolite. In fact, it has been found that the addition of Fe(III)-zeolite Y or Fe(II)-zeolite Y without H_2_O_2_ does not evidence any change in the UV-Vis spectra of the dye solutions, even after 24 h of treatment.  Figure 5 shows the UV-Vis spectra of the AR 14 solution for different contact time under heterogeneous Fenton-like process after the addition of H_2_O_2_ (see [25] for heterogeneous Fenton process). The absorption of the main peak decreased as the reaction time increased to finally disappear indicating the degradation of the dye.

**Figure 6:**
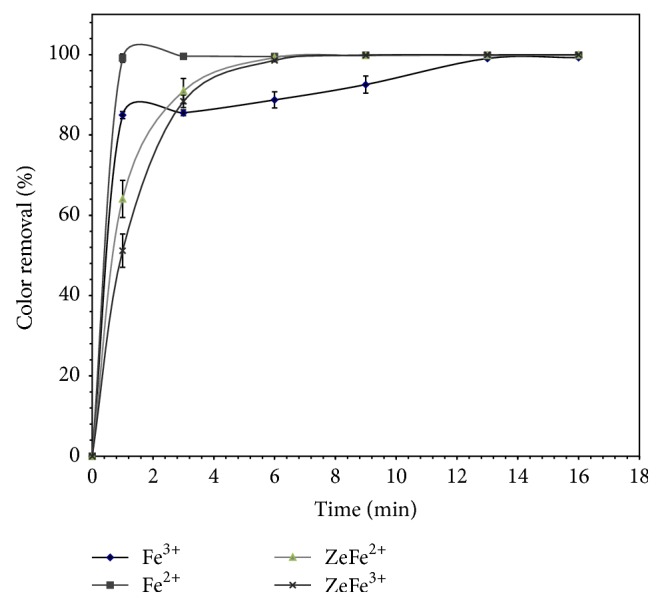
Color removal with time for the processes Fe^2+^/H_2_O_2_, Fe^3+^/H_2_O_2_, Fe(II)-Y zeolite/H_2_O_2_, and Fe(III)-Y zeolite/H_2_O_2_ at optimized parameters.

Figure 6 shows the variation of the dye decolorization for the four systems under study Fe^2+^/H_2_O_2_, Fe^3+^/H_2_O_2_, Fe(II)-zeolite Y/H_2_O_2_, and Fe(III)-zeolite Y/H_2_O_2_ at the optimized parameters. The yield of the dye decolorization was higher than 99% after 13 min for all the processes. At the beginning, the decolorization rate in homogeneous Fenton was much faster than in Fenton-like reaction (apparent rate constants *k* = 5.103 and 1.9068 min^−1^, resp., calculated by (5)). This fact is probably due to the additional step involved in the Fenton-like system: the conversion of Fe^3+^ into Fe^2+^ to generate the free radicals. For the heterogeneous systems, slight difference was only observed at the beginning of the reaction (*k* = 0.9051 for Fe(II)-zeolite Y/H_2_O_2_ and 0.6935 min^−1^ for Fe(III)-zeolite Y/H_2_O_2_). After a short period of three minutes, the degree of decolorization was similar for both processes (99% after 9 min).

### 3.3. Mineralization and Microtox Analysis

TOC was measured for the initial dye solution and after 15 min of treatment for the homogenous and heterogeneous Fenton processes at the established conditions. The results of TOC removal of AR 14 are shown in Table 1. From these results, it is deduced that the mineralization of AR 14 proceeds slower than the decolorization, result that is in accordance with a dye bath degradation study by electrochemical treatment [4]. By this last technique, the color of a reactive dye solution (1 g/L) was totally removed after 90 min of treatment but dye mineralization was much slower, with 10 h of electrochemical treatment being necessary to reach a TOC reduction of 81%. This difference between decolorization and mineralization is attributed to the extent of the reactions. In the first case, the decolorization is simply achieved by breaking the azo linkages of the dye. However, the mineralization is reached when the organic matter has been fully destroyed to produce CO_2_ and H_2_O. Because of this, the technique is proposed as a pretreatment to be carried out only on the colored waste-water, which must be segregated and decolorized previously to its incorporation to the biological treatment, combination that showed being very effective for the removal of color and organic matter [28].

The inhibitory effects produced by the different treatments on the bioluminescence of* Vibrio fischeri* were studied as previously described. For the different treatments, results of EC_50_, TII_50_, and blank expressed as TII_50_ demonstrated that the inhibitory effects due to the dye oxidation by-products were very low. The only contribution to the contamination is the chemicals introduced by the process itself because the system under consideration showed in all cases nonsignificant differences with respect to the blanks (Table 1). The only exception found was for the Fenton-like system which did not show toxicity. To better understand the origin of the toxicity, new experiments with different amounts of zeolite (2.5, 15.0, and 30.0 g/L) were carried out and results did not show any toxic effect. On the other hand, a solution containing 15.0 g/L of zeolite with 8.7 mM of H_2_O_2_ was also analyzed. The TII_50_ showed a value of 43.5 which increased to 125 when the same amount of H_2_O_2_ without zeolite was analyzed, meaning that the toxicity was coming exclusively by the remaining hydrogen peroxide and that the heating was not sufficient to completely eliminate it after the treatment. All the experiments were always performed in the same way as the heterogeneous experiment previously described.

### 3.4. Leaching and Stability Tests for the Heterogeneous Processes

During the process, iron ions can be released from the zeolite to the solution generating a secondary pollution and degrading the catalyst performance. In order to measure the amount of iron released, after each experiment the solution was filtered and analyzed by ICP-OES. The concentration of the iron ions released to the solution was found to be 0.2 ± 0.1 ppm and 0.6 ± 0.2 ppm. These results show that the decolorization and mineralization are mainly due to the heterogeneous catalyst activity (Fe(II)-zeolite Y/H_2_O_2_ and Fe(III)-zeolite Y/H_2_O_2_) and not to the leached iron ions.

Another important property of a catalyst is its long-term stability. In order to assess the catalytic activity of the zeolite during successive experiments, zeolite recovered by filtration from the solution after the treatment was washed with MilliQ water, dried at 60°C (overnight), and then tested again under the same reaction conditions. The color removal percentage for the three consecutive cycles was 99.96 ± 0.01, 99.8 ± 0.1, and 93 ± 1 for Fe(II)-zeolite Y/H_2_O_2_ and 99.77 ± 0.04, 98.5 ± 0.5, and 94 ± 1 for Fe(III)-zeolite Y/H_2_O_2_ catalytic studies. As it is seen, when the zeolite was reused three consecutive times, its catalytic activity was still higher than 90%, indicating a very low deactivation, which could be produced by the release of iron to the media or by deactivation of the catalyst.

## 4. Conclusions

In the present work, TOC and Microtox have been used to complement the information obtained after Fenton and Fenton-like processes for mineralization and toxicity, respectively. The homogeneous treatment with Fe^2+^/H_2_O_2_ is very effective for the complete removal of color after 3 min and only 15 minutes is needed to get a mineralization higher than 90%. On the other hand, the heterogeneous process achieves total decolorization in 6 minutes with a mineralization higher than 84%, with the mineralization of the dye, in all cases, being slower than the decolorization. The catalyst can be recovered and reused at least three times as the iron is not released to the solution avoiding the generation of the nondesired sludge, from an economical and environmental point of view, which increases the overall costs of the Fenton process. Another great advantage of zeolite Y process versus homogeneous Fenton process is the operation pH range. Whereas in the homogeneous process a strong acid pH value is required, in the heterogeneous one it is possible to work at a wider pH range which is very convenient because effluents may present a wide range of pH depending on the type of dyes used, thus reducing the costs of pH adjusting before discharge and associated problems. Microtox analysis showed that for all the processes the low toxicity generated was produced only by the residual H_2_O_2_.

## Figures and Tables

**Table 1:** Results for the mineralization and toxicity tests.

Sample	TOC	EC_50_	TII_50_ (Equitox/m^3^)	Blank TII_50_
Fe^3+^/H_2_O_2_	73 ± 7%	Nontoxic		
Fe^2+^/H_2_O_2_	91 ± 4%	6 ± 3%	20 ± 11	28
Fe(III)-zeolite Y/H_2_O_2_	86 ± 1%	2.3 ± 0.3%	45 ± 6	48
Fe(II)-zeolite Y/H_2_O_2_	84 ± 6%	24 ± 8%	4 ± 1	7
